# Pro‐apoptotic effect of haem oxygenase‐1 in human colorectal carcinoma cells via endoplasmic reticular stress

**DOI:** 10.1111/jcmm.14482

**Published:** 2019-06-14

**Authors:** Ming‐Shun Wu, Chih‐Chiang Chien, Jungshan Chang, Yen‐Chou Chen

**Affiliations:** ^1^ Division of Gastroenterology, Department of Internal Medicine Wan Fang Hospital, Taipei Medical University Taipei Taiwan; ^2^ Division of Gastroenterology and Hepatology, Department of Internal Medicine, School of Medicine, College of Medicine Taipei Medical University Taipei Taiwan; ^3^ Department of Nephrology Chi‐Mei Medical Center Tainan Taiwan; ^4^ Department of Food Nutrition Chung Hwa University of Medical Technology Tainan Taiwan; ^5^ Graduate Institute of Medical Sciences, College of Medicine Taipei Medical University Taipei Taiwan; ^6^ Cancer Research Center and Orthopedics Research Center, Taipei Medical University Hospital Taipei Taiwan; ^7^ Cell Physiology and Molecular Image Research Center Wan Fang Hospital, Taipei Medical University Taipei Taiwan

**Keywords:** apoptosis, CoPP, ER stress, haem oxygenase‐1, human colorectal carcinoma cells

## Abstract

Several biological effects of haem oxygenase (HO)‐1, including anti‐inflammatory, antiapoptotic and antioxidative properties were reported; however, the role of HO‐1 in apoptosis is still unclear. In the presence of stimulation by cobalt protoporphyrin (CoPP), an HO‐1 inducer, apoptotic characteristics were observed, including DNA laddering, hypodiploid cells, and cleavages of caspase (Casp)‐3 and poly(ADP) ribose polymerase (PARP) proteins in human colon carcinoma COLO205, HCT‐15, LOVO and HT‐29 cells in serum‐free (SF) conditions with increased HO‐1, but not heat shock protein 70 (HSP70) or HSP90. The addition of 10% foetal bovine serum (FBS) or 1% bovine serum albumin accordingly inhibited CoPP‐induced apoptosis and HO‐1 protein expression in human colon cancer cells. CoPP‐induced apoptosis of colon cancer cells was prevented by the addition of the pan‐caspase inhibitor, Z‐VAD‐FMK (VAD), and the Casp‐3 inhibitor, Z‐DEVD‐FMK (DEVD). N‐Acetyl cysteine inhibited reactive oxygen species‐generated H_2_O_2_‐induced cell death with reduced intracellular peroxide production, but did not affect CoPP‐induced apoptosis in human colorectal carcinoma (CRC) cells. Two CoPP analogs, ferric protoporphyrin and tin protoporphyrin, did not affect the viability of human CRC cells or HO‐1 expression by those cells, and knockdown of HO‐1 protein expression by HO‐1 small interfering (si)RNA reversed the cytotoxic effect elicited by CoPP. Furthermore, the carbon monoxide (CO) donor, CORM, but not FeSO_4_ or biliverdin, induced DNA ladders, and cleavage of Casp‐3 and PARP proteins in human CRC cells. Increased phosphorylated levels of the endoplasmic reticular (ER) stress proteins, protein kinase R‐like ER kinase (PERK), and eukaryotic initiation factor 2α (eIF2α) by CORM and CoPP were identified, and the addition of the PERK inhibitor, GSK2606414, inhibited CORM‐ and CoPP‐induced apoptosis. Increased GRP78 level and formation of the HO‐1/GRP78 complex were detected in CORM‐ and CoPP‐treated human CRC cells. A pro‐apoptotic role of HO‐1 against the viability of human CRC cells via induction of CO and ER stress was firstly demonstrated herein.

## 1 INTRODUCTION

Reactive oxygen species (ROS) are major cellular oxidants generated as byproducts of oxygen metabolism. Under some circumstances, ROS generation is greatly provoked by extracellular insults such as ionizing radiation, UV light, xenobiotics and pathogens, leading to an imbalance in the intracellular reduction‐oxidation status. Excessive levels of ROS can induce oxidative damage to DNA leading to gene mutations and carcinogenesis. Moreover, ROS may damage cellular structures and induce lipid peroxidation, eventually inducing apoptosis of various cells.^1^, ^2^ Clinically, ROS augmentation is a useful approach for cancer treatment, and various chemotherapeutic agents, such as cisplatin, nocodazole, and taxol, were shown to exert their antitumour activities through activating ROS‐dependent apoptosis in different tumour cells.^3^, ^4^ Both pro‐survival and pro‐apoptotic actions by ROS overproduction have been demonstrated. Additionally, increased intracellular ROS levels as a proliferative signal were reported to promote the proliferation and survival of malignant cancer cells. The effects of reducing ROS levels on decreasing the viability of cancer cells are still unclear.

Haem oxygenase (HO)‐1 is a phase II enzyme that responds to oxidative stress, cellular injury and diseases by metabolizing haem into biliverdin (BV)/bilirubin (BR), carbon monoxide (CO) and ferrous iron.^5^ HO‐1 is regarded as a survival molecule, as it exerts cytoprotection against various cells in response to stressful conditions.^6^, ^7^, ^8^ HO‐1 is widely recognized to overcome assaults by augmented oxidative stress from chemotherapeutic agents to prevent cancer cells from undergoing apoptosis and even stimulating cell proliferation. Both protective and detrimental effects of HO‐1 were also reported in different diseases, including kidney injury and neurodegeneration.^9^, ^10^ Increasing evidence has shown a dark side of HO‐1, as it acts as a critical mediator in ferroptosis and as causative factor in the progression of several human diseases.^5^ Elevated HO‐1 levels were detected in various human malignancies, indicating its contribution to cancer cell growth, metastasis, and resistance to chemotherapy.^11^, ^12^ In contrast, augmented HO‐1 expression enhanced the death of many cancer cells.^13^, ^14^ Emerging evidence suggests another dark side of HO‐1 via inducing ferroptosis through iron accumulation. Although the bright and dark sides of HO‐1 have been discussed in different studies, the mechanism by which HO‐1 augmentation causes protective and cytotoxic activities in cancer cells is still unknown.

Colorectal cancer (CRC) is one of the leading diagnosed cancers with high mortality, and remains a significant global health problem. Many chemotherapeutic agents, such as taxol and carboplatin, are used to treat CRC; however, there are side effects with chemotherapy that are associated with high mortality and local recurrence at least in part through ROS production. In humans, haem‐iron is more bioavailable than non‐haem‐iron, and unabsorbed haem reaches colon epithelial cells.^15^ Previous studies showed that haem is able to irritate the epithelium of the colon as indicated by mild diarrhoea.^16^, ^17^ Feeding haem resulted in significantly increased proliferation of colonic mucosa of rats.^18^ This indicates the positive correlation between haem and colon carcinogenesis. HO‐1 induction was shown to metabolize haem, accompanied by producing four byproducts: CO, ferric ion, BV and BR The effects of HO‐1 overexpression on CRC treatment and the roles that ROS and their byproducts play in the process are still unclear.

Cobalt protoporphyrin (CoPP) is a substrate for HO and was identified as a potent HO‐1 inducer.^19^ Previous studies indicated that CoPP is able to increase endogenous CO generation against myocardial infarction in vivo, and decrease production of inflammatory molecules in the central nervous system.^20^, ^21^ In microglia, CoPP protected against lipopolysaccharide (LPS) interleukin‐13‐induced apoptosis and reduced the expression of monocyte chemoattractant protein‐1 and microglia recruitment in a retinal injury mouse model. Our previous study demonstrated that CoPP inhibited LPS‐ or lipoteichoic acid (LTA)‐induced inducible nitric oxide (NO) synthase (iNOS) and NO production by microglia.^22^ Although several biological activities of HO‐1 induced by CoPP have been reported, the effect of HO‐1 on the viability of human colon carcinoma cells is still undefined. In the present study, we found that CoPP preferentially reduced the viability of three poorly differentiated CRC cell lines, COLO205, HCT‐15 and LOVO, but had less of an effect on the well‐to‐modestly differentiated colon carcinoma HT‐29 cells. The roles of HO‐1 protein, ROS production, CO and ER stress in CoPP‐induced apoptosis of human CRC cells are investigated in the present study.

## 2 MATERIALS AND METHODS

### 2.1 Cells

Human CRC cell lines, including COLO205 (ATCC^®^ CCL‐222^™^)), HCT‐15 (ATCC^®^ CCL‐225^™^), LOVO (ATCC^®^ CCL‐229^™^) and HT‐29 (ATCC^®^ HTB‐38^™^) were sourced from American Type Culture Collection (Manassas, VA). WI‐38 cells (ATCC^®^ CCL‐75™), a diploid human cell strain derived from normal lung tissue of a 3‐month gestation aborted female foetus, were obtained from ATCC, and NHLF (Catalog #: CC‐2512) was purchased from LONZA. Cells in Dulbecco's modified Eagle medium (DMEM) containing 10% heat‐inactivated foetal bovine serum (FBS; Gibco/BRL, Grand Island, NY), supplemented with antibiotics (100 U/mL penicillin A and 100 U/mL streptomycin) were maintained in a 37°C humidified incubator containing 5% CO_2_. Cells were used between passages 18 and 30 for all experiments. After reaching confluence, cells were seeded onto 6‐cm dishes for further experiments.

### 2.2 Materials

Cobalt protoporphyrin, ferric protoporphyrin (FePP), tin protoporphyrin (SnPP), nitroblue tetrazolium (NBT), curcumin, FeSO_4_, CORM, RuCL_3_ and 5‐bromo‐4‐chloro‐3‐indolyl phosphate (BCIP) were purchased from Sigma (St. Louis, MO). All chemicals were dissolved in dimethyl sulfoxide (DMSO), and the final concentration of DMSO in each treatment was <0.5%. Antibodies of total PERK (tPERK; sc‐32577), HO‐1 (sc‐7695) and α‐tubulin (α‐Tub; sc‐5286) were obtained from Santa Cruz Biotechnology (Santa Cruz, CA). Antibodies of phosphorylated PERK (Thr980; pPERK; #5683), poly(ADP) ribose polymerase (PARP; #9542), and binding immunoglobulin protein (BiP)/Grp78 proteins were obtained from Cell Signaling Technology (Beverly, MA). Antibody of caspase‐3 protein (Img‐144A) was purchased from IMGENEX (San Diego, CA). Antibodies of HSP70 (#610608), HSP90 (#610419), were obtained from BD Biosciences. Antibodies of total eIF‐2α (teIF‐2α; ab32157), phosphorylated eIF‐2α (Ser51; peIF‐2α; ab32157) were obtained from abcam (Eugene). Small interfering (si)RNA of HO‐1 and control siRNA, were obtained from Santa Cruz Biotechnology.

### 2.3 Western blotting

Cells lysates were prepared by suspending cells in RIPA buffer, and equal amounts of protein were prepared and separated on sodium dodecylsulfate‐polyacrylamide mini gels and transferred to Immobilon polyvinylidene difluoride membranes (Millipore, Bedford, MA). Membranes were incubated at 4°C with 1% bovine serum albumin (BSA) for a further 50 minutes at room temperature and then incubated with the indicated antibodies overnight at 4°C. Followed by incubation with an alkaline phosphatase‐conjugated immunoglobulin G antibody for 1 hour. Proteins were visualized by incubating with the colorimetric substrates, NBT and BCIP.

### 2.4 MTT (3‐(4,5,‐dimethylthiazol)‐2‐yl‐2,5‐diphenyltetrazolium bromide) assay

Cells were plated at a density of 5 × 10^4^ cells/well in 24‐well plates. At the end of treatment, the supernatant was removed, and 30 μL of the tetrazolium compound, MTT, and 270 mL of fresh DMEM were added. After incubation for 2 hours at 37°C, 200 μL of 0.1 N HCl in 2‐propanol was placed in each well to dissolve the tetrazolium crystals. Finally, the absorbance at a wavelength of 600 nm was recorded using an enzyme‐linked immunosorbent assay (ELISA) plate reader.

### 2.5 In vitro morphology

Human CRC cells were grown at a density of 105 cells/well in 24‐well plates for 24 hours. Cells were treated with or without CoPP, and cells were fixed with 3.7% formaldehyde. Cell morphological changes were examined under a light microscope.

### 2.6 DNA fragmentation assay

Cells under different treatments were collected, and then lysed in 100 µL of lysis buffer (50 mmol/L Tris at pH 8.0, 10 mmol/L ethylenediaminetetraacetic acid (EDTA), 0.5% sodium sarkosinate, and 1 mg/mL proteinase K) for 3 hours at 56°C. Then, 0.5 mg/mL RNase A was added to each reaction for another hour at 56°C. DNA was extracted with phenol/chloroform/isoamyl alcohol (25/24/1) before loading. Then, DNA samples were mixed with 6 µL of loading buffer (50 mmol/L Tris, 10 mmol/L EDTA, 1% (w/w), and 0.025% (w/w) bromophenol blue), and loaded onto a 2% agarose gel containing 0.1 mg/mL ethidium bromide. The agarose gels were run at 100 V for 45 minutes in TBE buffer, then observed and photographed under UV light.

### 2.7 Detection of hypodiploid cells by CoPP

Cells were plated in 24‐well plates in duplicate, then incubated for 24 hours. Media were removed, and different treatments were added to each well. Cells were treated for 12 hours, and the supernatant and cells were harvested by exposing cells to a 0.25% trypsin‐EDTA solution for 10 minutes, then centrifuged, washed in PBS, and fixed in 3 mL ice‐cold 100% ethanol. All samples were incubated for 30 minutes at room temperature in the dark. The cell cycle distribution and hypodiploid cells were determined using a FACSan flow cytometer (FACScan, Becton Dickinson).^23^

### 2.8 Measurement of ROS generation

Intracellular peroxide levels in the presence or absence of various treatments were measured by the oxidation of DCFH‐DA to DCF. DCFH‐DA is a non‐polar compound that readily diffuses into cells and is hydrolysed to the non‐fluorescent polar derivative, DCFH, which is trapped within cells, and the oxidized DCFH‐DA turns into the highly fluorescent DCF. Cells were incubated in the dark for 10 minutes at 37°C with 50 μmol/L DCFH‐DA, then resuspended in plain medium. The fluorescence was analysed using a FACScan (Becton Dickinson, Sunnyvale, CA) flow cytometer with excitation at 488 nm and emission at 530 nm.

### 2.9 Statistical analysis

Values are expressed as the mean ± SD of triplicate experiments. The significance of the difference from the respective controls for each experimental was assayed using a one‐way analysis of variance (ANOVA) with a post hoc Bonferroni analysis when applicable, and *P* < 0.05 or *P* < 0.01 were considered statistically significant.

## 3 RESULTS

### 3.1 The HO‐1 inducer, CoPP, reduced the viability of human CRC cells

**Figure 1:**
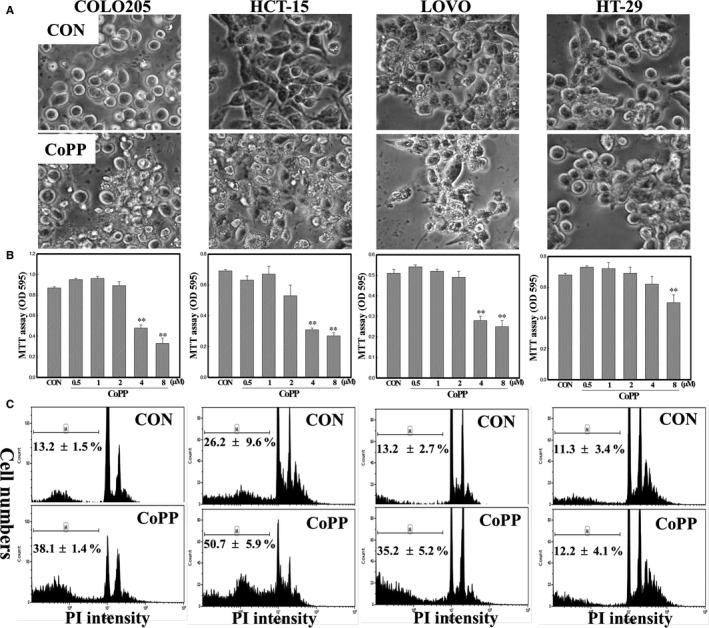
Cobalt protoporphyrin (CoPP) inhibition of viability of COLO205, HCT‐15, LOVO and HT‐29 human colorectal carcinoma cells. (A), Cells were treated with CoPP (8 µmol/L) for 12 h in a serum‐free (SF) condition, and morphological changes were examined under microscopic observation. (B), The viability of indicated cells was examined by an MTT assay. Cells were treated with different concentrations (0.5, 1, 2, 4 and 8 µmol/L) of CoPP for 12 h, and the viability of cells was examined by an MTT assay as described in ‘Materials and Methods’. (C), The percentage of hypodiploid cells (sub‐G_1_ peak) in CoPP‐treated human colorectal carcinoma cells was examined by a flow cytometric analysis using propidium iodide (PI) staining. Cells were treated as described in (A), and the percentage of hypodiploid cells in the indicated cells was detected by a flow cytometric analysis. Each data point was calculated from three triplicate groups, and data are shown as the mean ± SD. **P < 0.01 denotes a significant difference between indicated groups

In order to examine the role of HO‐1 in apoptosis of human CRC cells, HO‐1 inducer CoPP and four human colorectal carcinoma cell lines, including COLO205, HT‐29, HCT‐15 and LOVO were used in the study. As illustrated in Figure 1A, morphological changes induced by CoPP were detected in human CRC cells under microscopic observations in the absence of serum addition, and it appeared that CoPP treatment induced the occurrence of smaller rounded bodies (apoptotic bodies). Data of the MTT assay showed that decreased viability of human CRC cells was detected, and 50% inhibitory concentration (IC_50_) values for COLO205, LOVO, HCT‐15 and HT‐29 cells were 4.5 ± 0.7, 5.8 ± 0.5, 4.7 ± 0.3 and >8.0 µmol/L, respectively (Figure 1B). Data derived from a flow cytometric analysis using propidium iodide (PI) staining indicated that percentages of hypodiploid cells had increased in CoPP‐treated human CRC cells, and those levels were much higher in COLOL205, LOVO and HCT‐15 compared to HT‐29 cells (Figure 1C). We further examined that cytotoxicity of CoPP in normal cells, and two preferential normal cells including WI‐38 and normal human lung fibroblast (NHLF) were used. Data of MTT assay showed that human colorectal carcinoma cells COLO205 and HCT‐15 expressed high sensitivity to CoPP‐induced cytotoxicity, compared with WI‐38 and NHFL cells. (Figure S2).

### 3.2 CoPP's reduction in cell viability via apoptosis was associated with induction of HO‐1 protein expression in human CRC cells

**Figure 2:**
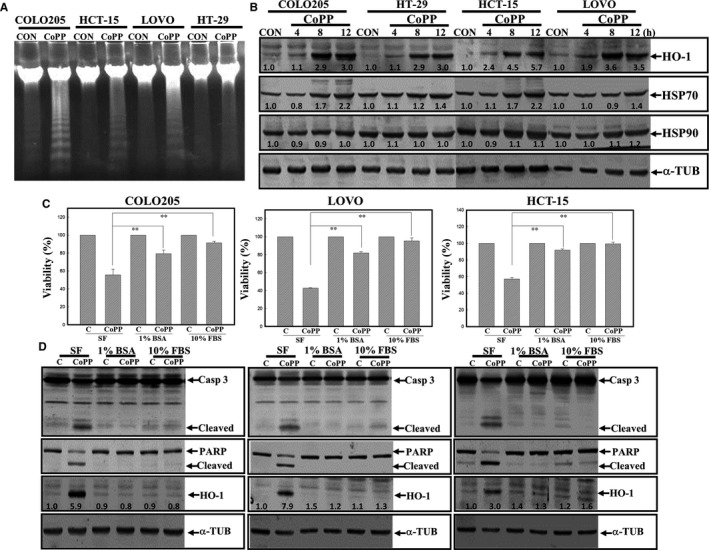
Cobalt protoporphyrin (CoPP) induction of apoptosis with increased Haem oxygenase (HO)‐1 protein expression in human colorectal carcinoma cells. (A), Induction of DNA ladders in CoPP‐treated colorectal carcinoma cells. Cells were treated with CoPP (8 µmol/L) for 12 h, and DNA integrity was analysed by agarose electrophoresis. (B), Increased HO‐1 protein expression was detected in CoPP‐treated human colorectal carcinoma cells. Cells were treated with CoPP (8 µmol/L) for different times (4, 8 and 12 h), and expressions of indicated proteins including HO‐1, heat shock protein 70 (HSP70), HSP90 and α‐tubulin (α‐TUB) were examined by Western blotting using specific antibodies. (C), CoPP exhibited potent cytotoxicity in a serum‐free (SF) condition. Cells were cultured in an SF condition with or without 1% bovine serum albumin (BSA) or 10% foetal bovine serum (FBS), followed by CoPP (8 µmol/L) treatment for 12 h. The viability of cells under different treatments was examined by an MTT assay. (D), As described in (C), expressions of apoptotic proteins such as caspase‐3 and poly(ADP) ribose polymerase (PARP), HO‐1 and α‐TUB were examined by Western blotting. The intensity of HO‐1, HSP70 and HSP90 protein was quantitated with normalization to α‐TUB by a densitometric analysis (ImageJ), and expressed as folds of control (C). Data from three independent experiments were obtained, and results are shown as the mean ± SD. **P < 0.01 denotes a significant difference between indicated groups

Data of the DNA integrity assay showed that CoPP induction of DNA ladders in human colorectal carcinoma cells and the DNA ladder intensity were higher in COLO205, HCT‐15 and LOVO cells than in HT‐29 cells (Figure 2A). Expressions of heat shock proteins (HSPs), including HSP32 (HO‐1), HSP70 and HSP90, in human CRC cells under CoPP treatment were examined by Western blotting using specific antibodies. As shown in Figure 2B, increased levels of HO‐1, but not HSP70 or HSP90, protein were observed in human CRC cells. We further examined the effects of serum (FBS) and albumin (BSA) on CoPP induction of HO‐1 and cell death in human CRC cells. As illustrated in Figure 2C, the addition of FBS or BSA significantly reduced the cytotoxicity of CoPP on the viability of human CRC cells in an SF condition. Data of Western blotting indicated that CoPP‐induced HO‐1 protein expression and cleavage of Casp‐3 and PARP proteins were suppressed by the addition of FBS or BSA in human CRC cells (Figure 2D).

### 3.3 The VAD and DEVD caspase inhibitors protected human CRC cells from CoPP‐induced cell death

**Figure 3:**
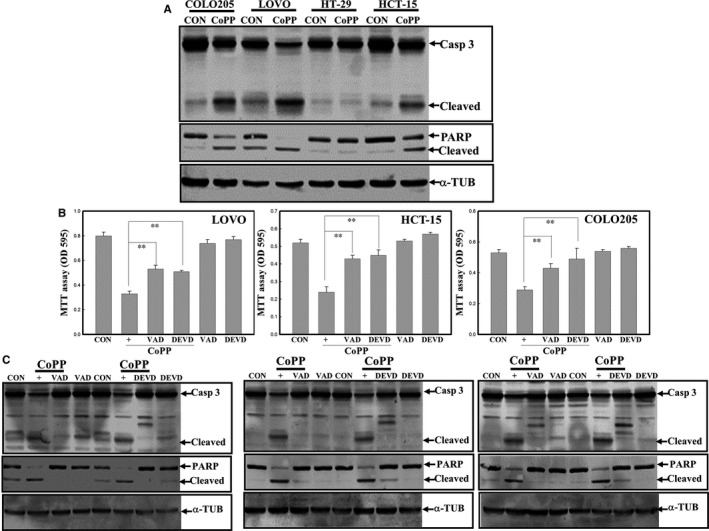
Activation of caspases participates in cobalt protoporphyrin (CoPP)‐induced apoptosis of human colorectal carcinoma cells. (A), Induction of caspase‐3 and poly(ADP) ribose polymerase (PARP) protein cleavage in CoPP‐treated COLO205, HCT‐15 and LOVO cells. (B), The pan‐caspase inhibitor Z‐VAD‐FMK (VAD) and the caspase‐3‐specific peptidyl inhibitor Z‐DVAD‐FMK (DVAD) inhibited CoPP‐induced cell death in colorectal carcinoma cells. Cells were treated with VAD or Z‐DEVD‐FMK (DEVD) (100 nmol/L) for 1 hour followed by CoPP (8 µmol/L) stimulation for 12 h, and the viability of indicated cells was examined by an MTT assay. (C), VAD and DEVD suppressed CoPP‐induced cleavage in the caspase‐3 and PARP proteins in LOVO, HCT‐15 and COLO205 cells. As described in (B), and expressions of indicated protein were examined by Western blotting. Data from three independent experiments were obtained, and results are shown as the mean ± SD. **P < 0.01 denotes a significant difference between indicated groups

We further examined the role of caspase activation in apoptosis by CoPP in human CRC cells. Two caspase inhibitors, including the pan‐caspase inhibitor, VAD, and the specific Casp‐3 inhibitor, DEVD, were used in the study. Data of Western blotting indicated that increased cleavage of Casp‐3 and PARP proteins were observed in COLO205, HCT‐15 and LOVO cells, but not in HT‐29 cells (Figure 3A). Results of the MTT assay in Figure 1B and Western blotting in Figure 3A suggested that HT‐29 cells were less sensitive to CoPP induction of apoptosis than were COLO205, HCT‐15 and LOVO cells. The addition of VAD or DEVD significantly reduced CoPP‐induced cell death in COLO205, HCT‐15 and LOVO cells (Figure 3B). Data of Western blotting indicated that VAD and DEVD inhibited CoPP‐induced cleavage of Casp‐3 and PARP proteins in these CRC cells (Figure 3C).

### 3.4 ROS did not participate in CoPP‐induced apoptosis of human CRC cells

**Figure 4:**
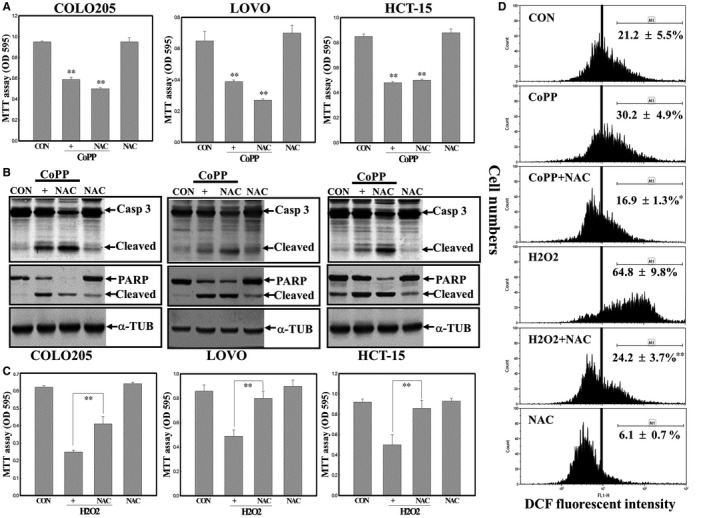
Reactive oxygen species (ROS) might not participate in cobalt protoporphyrin (CoPP)‐induced apoptosis of human colorectal carcinoma cells. (A), N‐acetyl cysteine (NAC) did not affect CoPP‐induced cell death of LOVO, HCT‐15 and COLO205 cells. Cells were treated with NAC (10 mmol/L) for 30 min followed by CoPP (8 µmol/L) stimulation for 12 h. The viability of indicated cells under different treatments was examined by an MTT assay. (B), NAC showed no effect on CoPP‐induced cleavage of caspase‐3 and poly(ADP) ribose polymerase (PARP) proteins in human colorectal carcinoma cells. As described in (A), protein expressions were examined by Western blotting. (C) NAC protected human colorectal carcinoma cells from oxidant H_2_O_2_‐induced cell death. As described in (A), H_2_O_2_ (100 µmol/L) was used to replace CoPP in the study. (D), NAC reduced peroxide levels in cells stimulated by H_2_O_2_ via a flow cytometric analysis. Cells were treated with NAC followed by H_2_O_2_ (100 µmol/L) or CoPP (8 µmol/L) treatment for 3 h, and intracellular peroxide levels were examined by a flow cytometric analysis using DCHFDA as a fluorescent dye. *P < 0.05, **P < 0.01 denote a significant difference from the CoPP‐ or H_2_O_2_‐treated group

We further examined if ROS production is involved in CoPP‐induced apoptosis of human CRC cells. The addition of the antioxidant NAC did not affect the decreased viability of COLO205, LOVO and HCT‐15 human CRC cells by CoPP according to the MTT assay (Figure 4A). Data of Western blotting showed that CoPP‐induced cleavage of Casp‐3 and PARP proteins was not altered by NAC in these three cell lines (Figure 4B). A positive study using H_2_O_2_ as an inducer of ROS‐dependent apoptosis indicated that NAC significantly reduced cell death by H_2_O_2_ in human carcinoma cells (Figure 4C). Intracellular peroxide levels were measured by a flow cytometric analysis using DCFHDA as a fluorescent probe, and data (Figure 4D) showed that intracellular peroxide levels were not altered by CoPP, but were increased by H_2_O_2_ in these three cell lines, and NAC showed an ROS‐scavenging effect thereby reducing peroxide levels in these cells (Figure 4D). These results suggested that CoPP‐induced apoptosis with increased HO‐1 protein expression was likely not related to ROS production in human CRC cells.

### 3.5 Increased HO‐1 protein expression contributes to CoPP‐induced cell death of COLO205, LOVO and HCT15 human CRC cells

**Figure 5:**
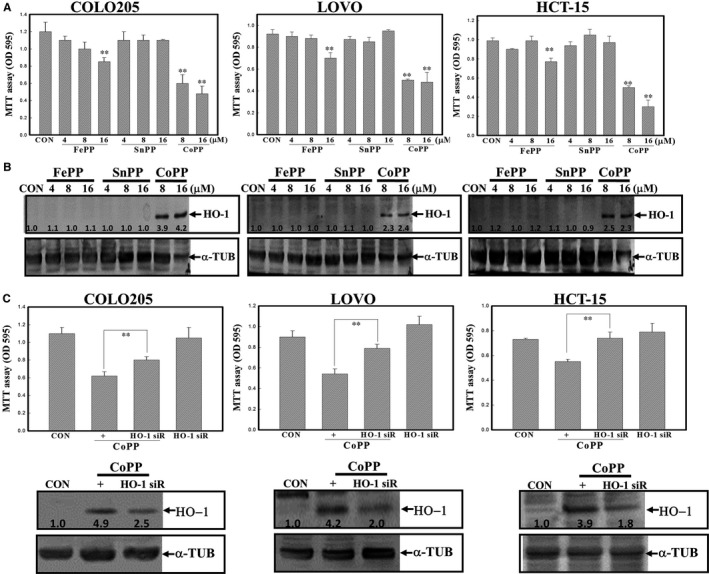
Haem oxygenase (HO)‐1 induction contributed to cobalt protoporphyrin (CoPP)‐induced cell death of human colorectal carcinoma cells. (A), CoPP exhibited the most potent cytotoxicity against human colorectal carcinoma cells. Three metal protoporphyrins including CoPP, ferric protoporphyrin (FePP) and tin protoporphyrin (SnPP) were used. Cells were treated with different concentrations (4, 8 and 16 µmol/L) of the indicated chemicals for 12 h, and the viability of indicated cells was examined by an MTT assay. (B), CoPP, but not FePP or SnPP, induced HO‐1 protein expression in LOVO, HCT‐15 and COLO205 cells. As described in (A), the level of the HO‐1 protein was examined by Western blotting. (C), Knockdown of HO‐1 protein expression reversed the cytotoxicity elicited by CoPP in human colorectal carcinoma cells. Cells were transfected with scrambled or HO‐1 siRNA for 24 h followed by CoPP (8 µmol/L) stimulation. The viability of indicated cells was examined by an MTT assay (upper panel), and decreased expression of the HO‐1 protein in HO‐1 siRNA‐treated cells was observed (lower panel). The intensity of HO‐1 protein was quantitated with normalization to α‐TUB by a densitometric analysis (ImageJ), and expressed as folds of control (C). Data from three independent experiments were obtained, and results are shown as the mean ± SD. **P < 0.01 denotes a significant difference from the control (CON) group (A) or between indicated groups (C)

There are three metal protoporphyrins, including FePP, zinc protoporphyrin (ZnPP) and CoPP, and they were reported to increase or decrease HO‐1 activity in various cells. Data of the viability assay showed that CoPP exhibited the strongest cytotoxic effect against the viability of human CRC cells (Figure 5A). Detection of HO‐1 protein expression in these three cell lines stimulated by FePP, ZnPP and CoPP showed that CoPP, but not the other two, induced HO‐1 protein in these cells according to the Western blot analysis (Figure 5B). Transfection of HO‐1 siRNA significantly reduced HO‐1 protein expression and cytotoxicity elicited by CoPP in COLO205, LOVO and HCT‐15 cells according to the MTT assay (Figure 5C).

### 3.6 Differential effects of HO‐1‐catalysed products including CO, Fe^+2^ and BV on the viability of human CRC cells

**Figure 6:**
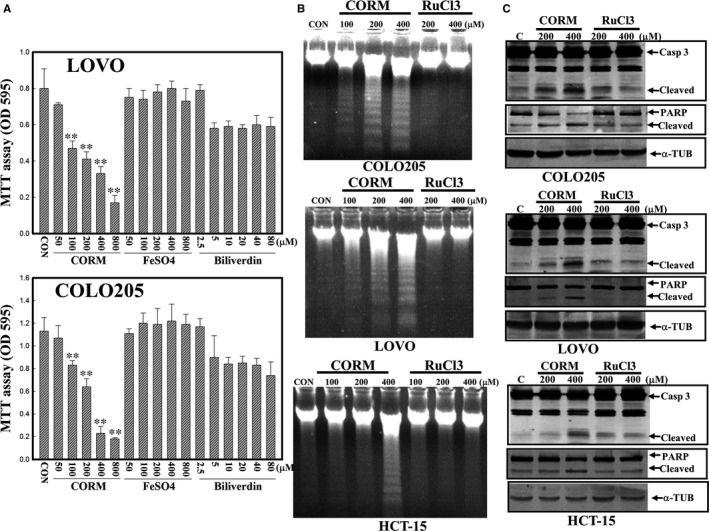
Carbon monoxide (CORM), but not FeSO_4_ or biliverdin (BV), caused a decrease in the viability of LOVO, HCT‐15 and COLO205 human colorectal carcinoma cells. (A), Both LOVO and COLO205 cells were treated with the indicated concentrations of CORM, FeSO_4_, or BV for 12 h, and the viability of cells was examined by an MTT assay. (B), CORM (RuCO) but not RuCl_3_ induced DNA ladders in human colorectal carcinoma cells. Cells were treated with indicated concentrations of CORM or RuCl_3_ for 12 h, and DNA integrity was examined by agarose electrophoresis. (C), CORM, but not RuCl_3_, induced cleavage in caspase‐3 and poly(ADP) ribose polymerase (PARP) proteins in LOVO, HCT‐15 and COLO205 cells. As described in (B), cleavage of the caspase‐3 and PARP proteins was examined by Western blotting. Data from three independent experiments were obtained, and results are shown as the mean ± SD. **P < 0.01 denotes a significant difference from the control (CON) group

Three products, including CO, Fe^+2^ and BV, were reported to be involved in haem metabolism by the HO‐1 protein; therefore we investigated the effects of these products on the viability of human CRC cells. Data of the MTT assay showed that CORM, but not Fe^+2^ or BV, concentration‐dependently decreased the viability of COLO205 and LOVO cells according to the MTT assay (Figure 6A). Analysis of DNA integrity by electrophoresis indicated that CORM (RuCO), but not its respective control compound RuCl_3_, induced DNA ladders in COLO205, LOVO and HCT‐15 cells (Figure 6B). Data of Western blotting showed that CORM, but not RuCl_3_, induced cleavage of Casp‐3 and PARP proteins in human CRC cells (Figure 6C).

### 3.7 Induction of ER stress by CoPP and CORM via formation of the HO‐1/BiP complex

**Figure 7:**
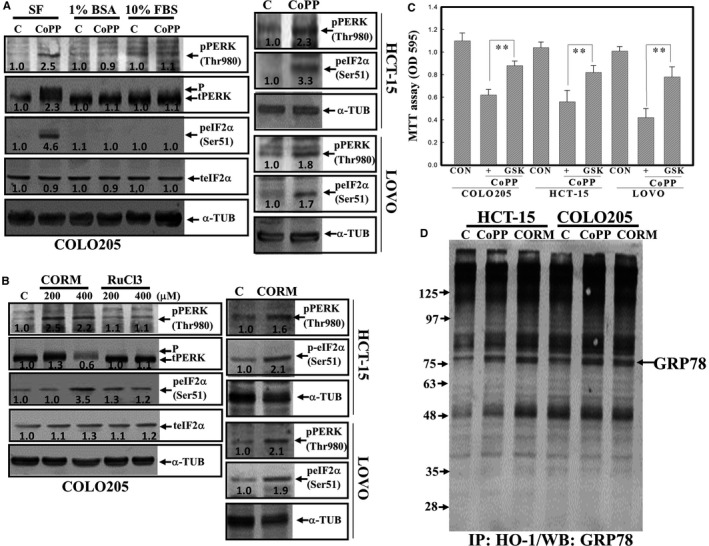
Cobalt protoporphyrin (CoPP) and carbon monoxide (CORM) induced expressions of endoplasmic reticular (ER) stress proteins, including phosphorylated protein kinase R‐like ER kinase (PERK) and eukaryotic initiation factor 2α (eIF2α) proteins in human colorectal carcinoma cells. (A), CoPP induction of the phosphorylated PERK protein at Thr 980 and phosphorylated eIF2α protein at Ser 51 in a serum‐free (SF) condition but not in the presence of bovine serum albumin (BSA) or foetal bovine serum (FBS) was detected by Western blotting using specific antibodies. Cells were treated with or without CoPP (8 µmol/L) under an SF, 1% BSA, or 10% FBA condition for 12 h, and expressions of indicated proteins were examined by Western blotting. (B), CORM, but not RuCl_3_, induced expression of phosphorylated PERK and eIF2α proteins in COLO205, LOVO and HCT‐15 cells. Cells were treated with different concentrations of CORM or RuCl_3_ for 12 h, and expressions of indicated proteins were examined by Western blotting. (C), The PERK inhibitor, GSK, inhibited CoPP‐ and CORM‐induced cell death in human colorectal carcinoma cells. Cells were treated with GSK (5 and 10 µmol/L) for 30 min followed by the addition of CoPP (8 µmol/L) or CORM (200 µmol/L) for 12 h, and the viability of cells under different treatments was examined by an MTT assay. (D), Formation of a haem oxygenase (HO)‐1/binding immunoglobulin protein (BiP) (GRP78) complex in CoPP‐ and CORM‐treated HCT‐15 and COLO205 cells. The HO‐1/BiP complex was examined by immunoprecipitation using an anti‐HO‐1 antibody followed by Western blotting using an anti‐BiP antibody. The intensity of indicated proteins was quantitated with normalization to α‐TUB by a densitometric analysis (ImageJ), and expressed as folds of control (C). Data from three independent experiments were obtained, and results are shown as the mean ± SD. **P < 0.01 denotes a significant difference from the control (CON) group

We further examined if CoPP and CORM affected expressions of ER stress proteins including PERK, eIF2α and BiP/GRP78 in human CRC cells. As shown in Figure 7A, CoPP in an SF condition induced the phosphorylation of the PERK protein at Thr980 and eIF2α at Ser51 in COLO205, LOVO and HCT‐15 human CRC cells. Similarly, the addition of CORM increased phosphorylation of the PERK protein at Thr980 and eIF2α at Ser51 in COLO205, LOVO and HCT‐15 human CRC cells (Figure 7B). The addition of the PERK inhibitor, GSK2606414 (GSK), inhibited CoPP or CORM‐induced cell death in COLO205, HCT‐15 and LOVO human CRC cells according to the MTT assay (Figure 7C and Data not shown). Results of co‐immunoprecipitation followed by a Western blot analysis showed that the HO‐1 protein was able to bind to the BiP/GRP78 protein in COLO205 and HCT‐15 cells under CoPP and CORM stimulation (Figure 7D).

## 4 DISCUSSION

We explored the apoptotic role of HO‐1 in human CRC cells in the present study. Haem irritates the epithelium of the colon, resulting in significantly increased proliferation of colonic mucosa of rats, which suggests a correlation between haem and colon cancer formation. The effects of HO‐1 overexpression in human colon carcinoma cancer cells are still unclear. We found that the HO‐1 inducer, CoPP, was able to reduce the viability of human CRC cells by increasing HO‐1 protein expression. Knockdown of HO‐1 protein expression by HO‐1 siRNA decreased HO‐1 protein levels, which were associated with reduced cytotoxicity in CoPP‐treated human CRC cells. Apoptosis elicited by CoPP was not affected by the addition of NAC, indicating that it occurred in an ROS‐independent manner. Among metabolites catalysed by HO‐1, the CO donor, CORM, exhibited apoptotic activity against the viability of human CRC cells, as did HO‐1 induction. Both CoPP and CORM‐induced activation of ER stress proteins, including PERK and eIF2α in cells, and data of IP‐Western blotting indicated that HO‐1 can form a complex with the ER stress initiator, BiP, in cells. The pro‐apoptotic activity of HO‐1 induction as related to CO and activation of ER stress in human CRC cells was demonstrated herein.

HO‐1 is involved in maintaining cellular homeostasis, and possesses cytoprotective and antiapoptotic activities by modulating oxidative damage. Induction of HO‐1 expression provides protection against apoptosis and DNA damage elicited by various stimuli. Under inflammatory stimulation, an increase in HO‐1 protein levels was able to reduce production of cytokines and NO by macrophages. Our previous studies demonstrated that overexpression of HO‐1 protected macrophages from H_2_O_2_‐induced apoptosis and LPS‐induced NO production.^24^, ^25^ However, the effect of HO‐1 induction itself on the viability of cancer cells is still unclear. We found that application of the HO‐1 inducer, CoPP, to colon carcinoma cells significantly reduced cell viability via apoptosis induction, as characterized by the occurrence of Casp‐3 and PARP cleavage. Knockdown of HO‐1 expression by siRNA inhibited CoPP‐induced cell death in CRC cells. We also compared the effect of metal protoporphyrins including FePP, SnPP and CoPP on the viability and HO‐1 protein induction in human carcinoma cells. Results showed that neither FePP nor SnPP induced HO‐1 protein or cytotoxicity in these cells. A positive correlation between HO‐1 induction and apoptosis of human CRC cells was indicated.

Reactive oxygen species production was identified as a mediator leading to apoptosis, and both ROS‐dependent and ‐independent apoptosis elicited by various agents was reported. Shen et al^26^ indicated quercetin's enhancement of arsenic‐induced apoptosis via stimulating ROS production in human HaCaT keratinocytes. Chow et al^19^ reported that increased ROS production contributed to FePP‐induced apoptosis of glioma cells. Ko et al^27^ revealed an ROS‐dependent mitochondrial pathway in the gossypol‐inhibited growth of human CRC cells. In order to clarify the role of ROS in apoptosis of human colon carcinoma cells by the HO‐1 inducer, CoPP, detection of intracellular peroxide levels and the addition of the antioxidant NAC were performed in the present study. It appeared that CoPP treatment did not affect intracellular peroxide levels, and NAC showed no protection against CoPP‐induced cell death in human CRC cells. H_2_O_2_ was used as a positive control, and increased peroxide levels and cell death by H_2_O_2_ were significantly inhibited by NAC. These results indicated that ROS likely do not participate in CoPP‐induced apoptosis of human CRC cells.

Haem oxygenase‐1 catalyses haem into BV, CO and free iron. Al‐Owais et al^28^ indicated that HO‐1 influences apoptosis via CO‐mediated inhibition of K^+^ channels. CO production was shown to participate in HO‐1 protection of SH‐SY5Y cells from Alzheimer's amyloid‐β‐induced toxicity. We examined the effects of three HO‐1‐catalysed metabolites, BV, CO (CORM) and free ion (FeSO_4_), on the viability of human CRC cells. Data indicated that CORM reduced the viability of cells with increased apoptosis characteristics including Casp‐3 and PARP protein cleavage. This suggested that HO‐1 inhibition of viability of human CRC cells was mediated, at least in part, by CO production.

The ER, an intracellular stress response induced by the accumulation of unfolded or misfolded proteins, and three sensors for ER stress, including protein kinase R‐like ER kinase (PERK), inositol‐requiring protein (IRE)‐1 and ATF6 have been identified.^29^ One of the major ER chaperones, BiP (GRP78), was able to bind to these sensors in non‐stressful conditions. In the response to ER stress, an increased level of the BiP/GRP78 protein was dissociated from the sensors and facilitated their activation via interactions with downstream proteins.^30^ Protecting cells from apoptosis and inducing cell apoptosis by ER stress have both been observed in various cells and systems. Best et al^31^ indicated ER stress‐mediated apoptosis in B‐cell lymphoma cells stimulated by ubiquitin‐activating enzyme inhibitors. PERK is a protein kinase located at the ER‐membrane, and accumulation of unfolded proteins leads to the dissociation of GRP‐78/BiP from PERK and induces the autophosphorylation of PERK protein. Our previous study showed that activation of PERK contributes to apoptosis elicited by evodiamine.^32^, ^33^ The HO‐1 protein is known to be localized to the ER, plasma membranes, mitochondria and nuclei, indicating specific compartments related to HO‐1's functions in eukaryotic cells. In the study, we found that CoPP and CORM‐induced phosphorylation PERK and its downstream eIF2α protein, indicating activation of ER stress in human colorectal carcinoma cells. Recent studies reported that ER stress attempt to increase the ability to degrade the misfolded proteins by upregulation of the ubiquitination‐proteasome machinery.^34^, ^35^ Data as shown in Figure S3, CoPP and CORM were able to induce ubiquitination of proteins in COLO205 and HCT‐15 cells. These results supported an occurrence of ER stress by CoPP and CORM in human colorectal carcinoma cells. Previous studies showed that excessive production of ROS caused the misfolding of proteins in the ER, which leads to ER stress.^36^, ^37^ It indicated that oxidative stress‐enhanced accumulation of misfolded proteins in the ER resulted in ER stress. However, Change et al (2018) reported that ROS‐independent ER stress promoted the stemness properties of cancer‐initiating cells.^38^ In the present study, CoPP treatment did not elevate the intracellular peroxide production via flow cytometric analysis using DCHFDA as a fluorescent dye, and addition of NAC showed no inhibition on CoPP‐induced HO‐1 and apoptosis in human colorectal carcinoma cells. Data of co‐immunoprecipitation assay showed that binding between HO‐1 and BiP/GRP78 proteins were observed in CoPP‐ or CORM‐treated human CRC cells. It suggested that CoPP‐induced ER stress might be through ROS‐independent manner, and formation of HO‐1/GRP78 complex to block the binding of GRP78 to ER stress sensors might participate in ER stress of human CRC cells under CoPP and CORM stimulation.

Colorectal cancer is a leading cause of cancer deaths and is among the most abundant cancer type that occurs in both sexes. It was shown that early stages of CRC are highly detectable and can be cured with surgical resection followed by standard therapy. However, survival rates in stage III and IV CRC patients decrease, and recurrence rates range 40%‐60% in the first 3 years. In the present study, four human CRC cell lines, including HT‐29 (Duke's type B), HCT‐15 (Duke's type C), COLO205 (Duke's type D) and LOVO, were used. It appeared that three human CRC cell lines (ie COLO205, LOVO and HCT‐15) in the late stages (Duke's C and D) possessed higher sensitivities to CoPP‐induced apoptosis than did early‐stage HT‐29 cells. Data of the flow cytometric analysis showed that both HCT‐15 and COLO205 cells expressed higher endogenous peroxide levels than did HT‐29 cells (Figure S1). This suggested that HO‐1 induction may potentially and preferentially treat late‐stage CRC at least in part due to higher endogenous peroxide levels related to malignancy of human CRC cells. Due to CoPP‐induced apoptosis and HO‐1 protein expression were suppressed by BSA or FBS addition, it indicating that CoPP might not be effective for in vivo study. Therefore, development of effective HO‐1 inducers deserved for verifying the anti‐CRC actions of HO‐1 in vivo.

## 5 CONCLUSIONS

To conclude, our results demonstrate that induction of HO‐1 contributes to apoptosis through activation of ER stress in human CRC cells. We found that poorly differentiated colorectal cells, such as COLO205, HCT‐15 and LOVO, showed greater sensitivity to apoptosis elicited by the HO‐1 inducer, CoPP, than did well‐differentiated HT‐29 cells. Data of the flow cytometric analysis revealed that endogenous peroxide levels in poorly differentiated COLO205 and HCT‐15 cells were higher than those in well‐differentiated HT‐29 cells. This indicated that increased HO‐1 protein preferentially induced apoptosis related to higher amount of endogenous ROS levels in poor/malignant human CRC. Our results may provide experimental basis for anti‐CRC effect of HO‐1 protein, and activation of ER stress is involved herein. Future studies using animal models are needed to assess the in vivo anti‐CRC actions of HO‐1.

## CONFLICT OF INTEREST

The authors declare that they have no conflict of interest.

## AUTHOR CONTRIBUTIONS

YC Chen and MS Wu conceived and designed the experiments. MS Wu, CC Chien and J Chang performed the experiments. MS Wu and CC Chien analysed the data. YC Chen and MS Wu wrote the paper.
