# Supplementary figures and images for: Pro‐apoptotic effect of haem oxygenase‐1 in human colorectal carcinoma cells via endoplasmic reticular stress
Source: J Cell Mol Med. 2019 Jun 14;23(8):5692–704. doi: 10.1111/jcmm.14482 (PMC6653387; doi:10.1111/jcmm.14482)

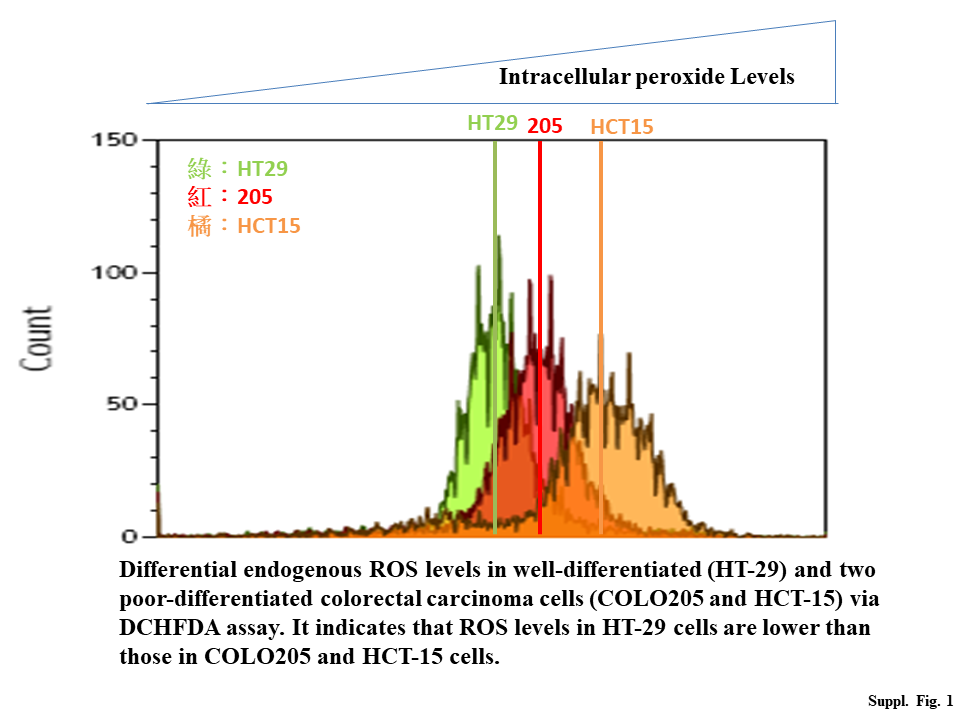

Supplement: Supplementary file 1 [file JCMM-23-5692-s001.TIF]

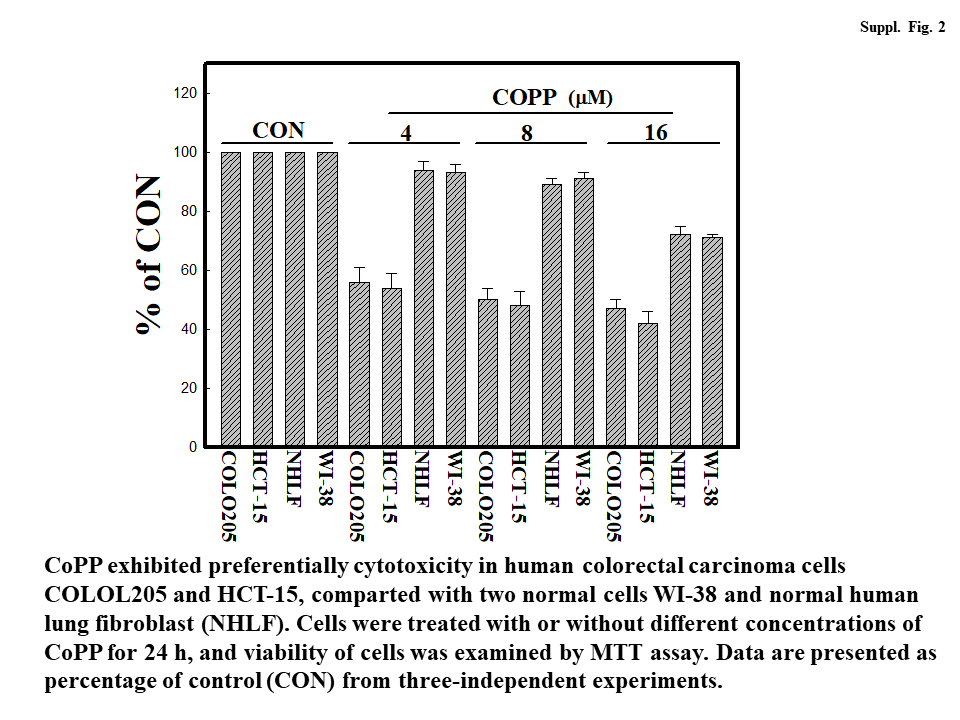

Supplement: Supplementary file 2 [file JCMM-23-5692-s002.TIF]

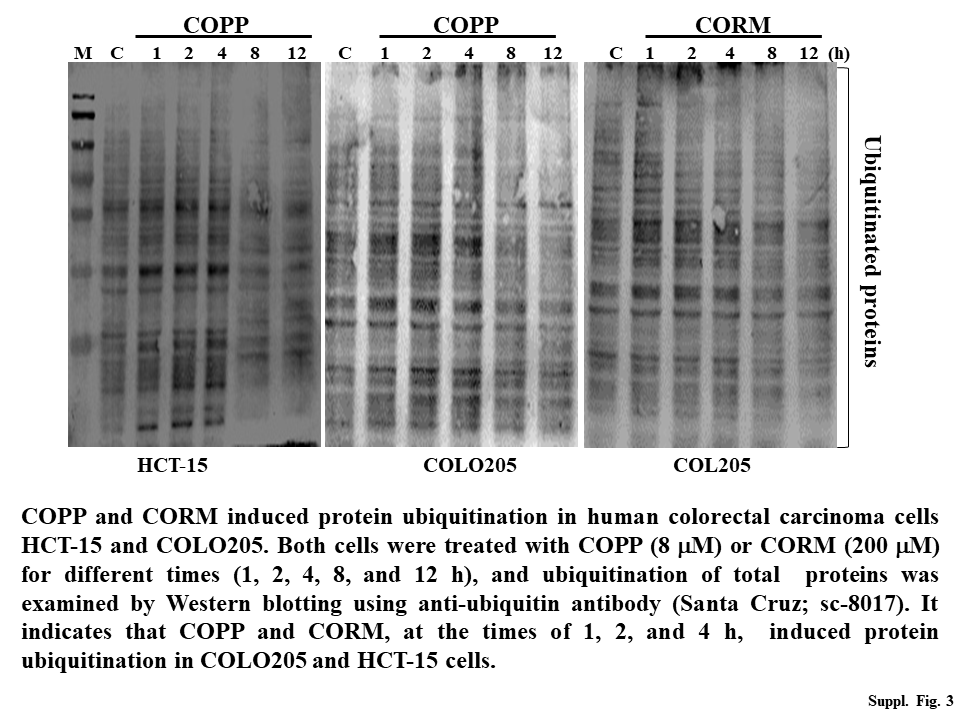

Supplement: Supplementary file 3 [file JCMM-23-5692-s003.TIF]
